# Real-world evidence for the impact of circadian clock on the benefit from immune checkpoint inhibitors in solid tumors

**DOI:** 10.1186/s12885-026-16364-w

**Published:** 2026-06-19

**Authors:** Eli M. Soyfer, Benjamin J. Lee, Abraham J. Qavi, Selma Masri, Angela G. Fleischman, Farshid Dayyani

**Affiliations:** 1 Division of Hematology/Oncology, Department of Medicine, School of Medicine, University of California, Irvine, CA USA; 2 Department of Biological Chemistry, School of Medicine, University of California, 839 Health Sciences Rd, Sprague Hall 126, Irvine, CA 92697 USA; 3 Department of Pharmacy, University of California, Irvine Health, Irvine, CA USA; 4 Department of Pathology & Laboratory Medicine, School of Medicine, University of California, Irvine, CA USA; 5 Sue and Ralph Stern Center for Cancer Clinical Trials and Research, University of California, Irvine, CA USA; 6 Chao Family Family Comprehensive Cancer Center, University of California, Irvine, CA USA

**Keywords:** Immune checkpoint inhibitors, Circadian rhythm, Disease control rate, Time-of-day, Real-world evidence

## Abstract

**Background:**

The circadian clock regulates tumor-immune interactions in a time-of-day-dependent manner, yet whether this translates to a clinically meaningful benefit across diverse solid tumors in real-world populations remains unclear.

**Methods:**

We retrospectively analyzed 969 patients receiving anti-PD-1 or anti-PD-L1 therapy at UCI Health between 2018 and 2022. Patients were classified as early (before 12 PM) or late (12 PM or after) based on infusion time. The primary outcome was disease control rate (DCR), defined as complete response (CR), partial response (PR), or stable disease (SD) per RECIST criteria. Unadjusted comparisons used the Pearson chi-square test. Multivariable logistic regression adjusting for disease stage, age, and ethnicity was performed in 806 patients with complete covariate data. Propensity score-matched analyses and sensitivity analyses using alternative exposure definitions were conducted.

**Results:**

Early infusion was associated with significantly higher DCR compared with late infusion (49% vs. 40%, *p* = 0.007). This association persisted after multivariable adjustment (adjusted OR 1.18, 95% CI 1.02–1.38, *p* = 0.024). The effect was directionally consistent across subgroups defined by sex, age, stage, and ICI agent. In ethnicity-stratified analyses, the association was significant among non-Hispanic patients (adjusted OR 1.18, 95% CI 1.02–1.39) but was indeterminate among Hispanic patients due to limited sample size (adjusted OR 1.18, 95% CI 0.84–1.65); the ethnicity-by-TOD interaction term was non-significant (*p* = 0.958). Sensitivity analyses using alternative exposure definitions and propensity score matching yielded consistent results.

**Conclusions:**

In a large, heterogeneous real-world cohort spanning multiple tumor types and a diverse patient population, early ICI administration was independently associated with higher DCR. These findings support a chronotherapeutic effect of ICI timing and underscore DCR as a sensitive endpoint for its detection. Prospective validation across tumor types and demographic subgroups is warranted.

**Supplementary Information:**

The online version contains supplementary material available at https://doi.org/10.1186/s12885-026-16364-w.

## Background

Immune checkpoint inhibitors (ICI) have transformed the treatment paradigm across multiple cancer types [1–3]. However, the presence of immune suppressive cells within the tumor microenvironment facilitates tumor growth via various mechanisms, ultimately leading to immune evasion of tumor cells and potentially influencing response to systemic treatments. An emerging field of study is the role of the circadian rhythm (CR) in affecting drug efficacy via regulation of immune suppressive cells based on the time of day of administration [4–6].

In preclinical models, the circadian clock is a key regulator of immune system activity within the tumor microenvironment. In mice bearing colorectal tumors, PD-L1–expressing MDSCs accumulate rhythmically, peaking during early waking hours, and anti-PD-L1 treatment yields the greatest antitumor responses when administered at the time of peak immune suppressive cell presence [5]. Other murine tumor models have revealed additional circadian dependencies in the antitumor immune response. Dendritic cell trafficking and T cell priming oscillate with CD80 expression [7], CD8 + T cell tumor infiltration and PD-1 expression fluctuate under the control of intrinsic leukocyte and endothelial cell clocks [8], and PD-1 on tumor-associated macrophages cycles diurnally through a DEC2-NF-κB axis [9]. Collectively, these findings demonstrate that independent circadian programs operating across multiple immune cell types converge to create time-of-day windows of optimal antitumor immunity, with immunotherapy most effective during the active phase in mice, corresponding to morning hours in diurnal humans.

Based on this preclinical rationale, several clinical studies have examined whether ICI administration timing affects patient outcomes. Retrospective analyses in individual cancer types, beginning with advanced melanoma [10] have generally supported a benefit of earlier ICI infusion [11–13]. The only prospective data come from a randomized phase 3 trial in non–small cell lung cancer (NSCLC) evaluating pembrolizumab plus platinum-based chemotherapy, which reported significantly longer progression-free survival (PFS) and overall survival (OS) with immunochemotherapy administered before 15:00 [14], however, questions have since been raised regarding protocol consistency in that trial. Critically, the literature lacks the breadth needed to guide prospective trial design, particularly in identifying the most appropriate disease sites, drug classes, and expected effect sizes for sample size calculations. To address these gaps, we retrospectively analyzed DCR by time of ICI administration at UCI Health from 2018 to 2022, across tumor types, regimens, and patient demographics.

## Methods

### Study design and data source

We conducted a retrospective cohort study of patients receiving ICI therapy at UCI Health between 2018 and 2022. Electronic medical records were queried to identify all patients with at least one documented ICI infusion with a recorded administration time. The study was approved by the UCI Institutional Review Board (UCI IRB #7327) in accordance with relevant guidelines and regulations, including the Declaration of Helsinki. All experiments were performed in accordance with the Declaration of Helsinki. Informed consent was waived given the retrospective observational design.

### Patients

Patients were included if they received at least one infusion of an anti-PD-1 or anti-PD-L1 agent and had a documented best response per RECIST criteria [15] following treatment. Patients without a recorded response status were excluded. Each patient contributed one record to the analysis; when multiple infusion time records were present, time-of-day group was assigned based on the first recorded infusion. Patients with any infusion before 12 PM were classified as early, and those with all infusions at or after 12 PM were classified as late.

### Exposure definition and cutoff justification

The 12 PM cutoff was selected based on its use in prior published ICI timing studies as a clinically meaningful threshold reflecting the transition from morning to afternoon infusion scheduling. Specifically, Patel et al. used the proportion of infusions administered before noon as the primary exposure definition in a multicenter mRCC cohort [16], and Karaboué et al. demonstrated that the median infusion time (approximately 12:54) effectively stratifies patients by outcome in NSCLC [17], supporting noon as a biologically and operationally relevant inflection point. Furthermore, a study-level meta-analysis by Landré et al. used 12:00–14:00 as the median-infusion-time cutoff across included studies [18]. The 12 PM threshold also aligns with standard morning and afternoon infusion slot scheduling at UCI Health.

### Outcomes

The primary outcome was DCR, defined as best response of CR, PR, or SD versus progressive disease (PD) or death per RECIST criteria as best response. Infusion time groups were dichotomized as before 12 PM (early) versus 12 PM or after (late).

### Covariates

Stage at initial diagnosis was extracted from the medical record and categorized as I through IV. Age was recorded at time of treatment initiation. Ethnicity was classified as Hispanic or non-Hispanic based on patient self-report in the medical record. ICI agent was classified as pembrolizumab, nivolumab, atezolizumab, or durvalumab. Line of therapy was recorded as the treatment regimen number in which ICI was administered.

### Statistical analysis

Differences in DCR between infusion time groups were assessed using the Pearson chi-square test without continuity correction. Proportions are presented with 95% confidence intervals (CIs) calculated using the Wald method. To assess whether the association between infusion time and DCR was independent of potential confounders, we performed multivariable logistic regression with early versus late infusion as the primary predictor and disease stage, age, and ethnicity as prespecified covariates; patients with missing data on any covariate were excluded from the adjusted model (*n* = 806). Results are reported as odds ratios (ORs) with 95% CIs. Propensity score-matched analyses were performed using 1:1 nearest-neighbor matching without replacement on stage, age, and ethnicity, with a caliper of 0.2 standard deviations of the logit propensity score; DCR in the matched cohort (*n* = 472) was compared using the chi-square test. Sensitivity analyses using three alternative exposure definitions (majority of infusions before noon, first infusion only, and median infusion time) were conducted to assess robustness of the primary exposure classification. To assess whether ethnicity modified the TOD-DCR association, we fit an interaction model including an ethnicity-by-TOD term and performed stratified adjusted analyses within each ethnicity subgroup. Site-specific effects were estimated using Fisher’s exact test for sites with *N* ≥ 20. Subgroup analyses by sex, age (age ≤ 65 vs. > 65), disease stage, and ICI agent were performed using chi-square tests and unadjusted ORs. OS was estimated using Kaplan–Meier methods and compared with the log-rank test; OS data are presented as supplementary material given stage imbalance between TOD groups. All analyses were performed in R (version 4.x; R Foundation for Statistical Computing, Vienna, Austria).

## Results

**Fig. 1:**
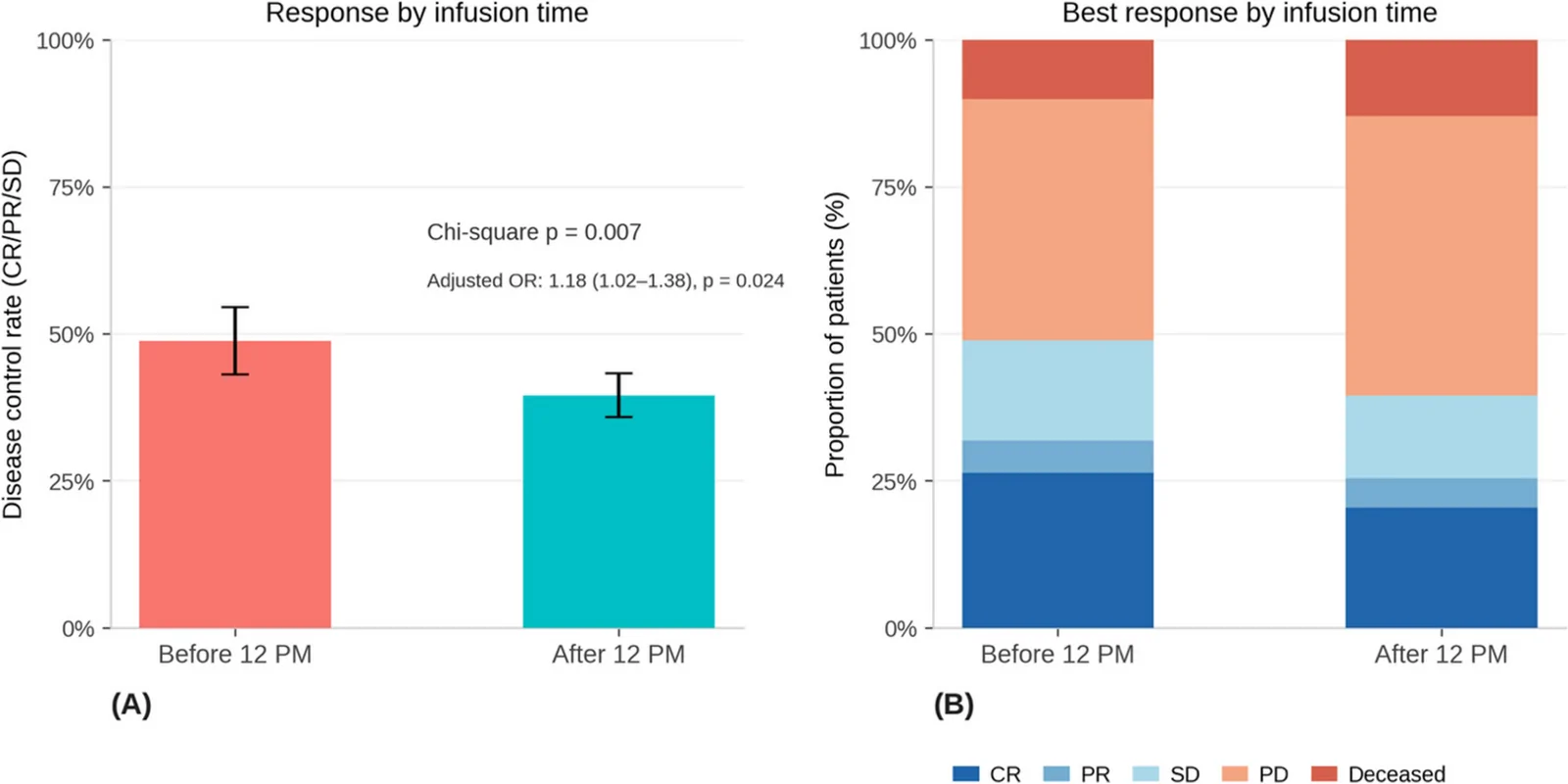
Association between time of ICI administration and clinical outcomes. **A **Disease control rate (DCR = CR/PR/SD) according to infusion time (before 12 PM vs. after 12 PM). Error bars indicate 95% confidence intervals (Wald method). P-value calculated using the Pearson chi-square test without continuity correction. Adjusted OR from multivariable logistic regression adjusted for stage, age, and ethnicity (*n* = 806). **B **Best response by infusion time shown as stacked proportions. CR = complete response; PR = partial response; SD = stable disease; PD = progressive disease; OR = odds ratio; CI = confidence interval

Of 1000 patients identified, treatment response was assessed using RECIST criteria [15], and those lacking a documented response status were excluded, yielding a final analytic cohort of 969 patients (Table 1). Patients were dichotomized into two cohorts: 293 (30%) received early (before 12 PM) and 676 (70%) received late (at or after 12 PM) infusions. This 2.3:1 ratio of late to early patients reflects real-world infusion scheduling patterns at our institution. The median age at treatment initiation was similar between early and late ICI groups (65.0 years [IQR 56.0–73.0] vs. 63.5 years [IQR 53.0–73.0]). The cohort was predominantly male (58%) and White (65%), with comparable sex and racial distributions between infusion-time groups. Most patients were non-Hispanic (78%), and the proportion of Hispanic patients was similar in the before- and after-12 PM cohorts (18% vs. 22%, respectively). At initial diagnosis, the majority of patients had stage IV disease (62%), although a higher proportion of stage IV cases was observed among the late ICI cohort (65% vs. 56%), whereas earlier-stage disease (stage I–II) was more common in the early ICI group (11% vs. 19%). Most patients received ICI in the third line or beyond (58%), with first-line receipt more common in the early cohort (25% vs. 19%). Pembrolizumab (65%) was the most frequently administered agent, followed by nivolumab (28%). Best response by category is shown in Table 1; Fig. 1B; patients treated before 12 PM had higher rates of CR (26% vs. 20%) and SD (17% vs. 14%) and lower rates of PD (41% vs. 48%).

**Table 1:** Baseline patient characteristics by time of immunotherapy administration

Characteristic	Overall *N* = 969	Before 12 PM *N* = 293	After 12 PM *N* = 676
Age at treatment, median (IQR)	64.0 (54.0, 73.0)	65.0 (56.0, 73.0)	63.5 (53.0, 73.0)
Sex			
Male	561 (58%)	159 (54%)	402 (59%)
Female	408 (42%)	134 (46%)	274 (41%)
Race			
White	631 (65%)	192 (66%)	439 (65%)
Asian	188 (19%)	61 (21%)	127 (19%)
Black	17 (1.8%)	8 (2.7%)	9 (1.3%)
Other/Unknown	133 (14%)	32 (11%)	101 (15%)
Ethnicity			
Hispanic	199 (21%)	52 (18%)	147 (22%)
Non-Hispanic	759 (78%)	239 (82%)	520 (77%)
Unknown/Refused	11 (1.1%)	2 (0.7%)	9 (1.3%)
Stage at initial diagnosis			
I	39 (4.8%)	18 (7.4%)	21 (3.7%)
II	70 (8.6%)	29 (12%)	41 (7.2%)
III	199 (25%)	61 (25%)	138 (24%)
IV	504 (62%)	136 (56%)	368 (65%)
Line of therapy			
First line	202 (21%)	72 (25%)	130 (19%)
Second line	198 (21%)	57 (20%)	141 (21%)
Third line or beyond	564 (58%)	161 (55%)	405 (60%)
ICI agent			
Pembrolizumab	630 (65%)	174 (59%)	456 (67%)
Nivolumab	272 (28%)	87 (30%)	185 (27%)
Atezolizumab	39 (4.0%)	20 (6.8%)	19 (2.8%)
Durvalumab	27 (2.8%)	11 (3.8%)	16 (2.4%)
Primary site			
HEENT	151 (16%)	48 (16%)	103 (15%)
Skin	139 (14%)	43 (15%)	96 (14%)
Lung	105 (11%)	34 (12%)	71 (11%)
Bladder	89 (9.2%)	19 (6.5%)	70 (10%)
Kidney	78 (8.0%)	15 (5.1%)	63 (9.3%)
Gynecologic	65 (6.7%)	19 (6.5%)	46 (6.8%)
Stomach	65 (6.7%)	33 (11%)	32 (4.7%)
Brain	52 (5.4%)	8 (2.7%)	44 (6.5%)
Colon/Rectum	45 (4.6%)	11 (3.8%)	34 (5.0%)
Liver	44 (4.5%)	7 (2.4%)	37 (5.5%)
Esophagus	34 (3.5%)	21 (7.2%)	13 (1.9%)
Breast	24 (2.5%)	13 (4.4%)	11 (1.6%)
Other/Unknown	44 (4.5%)	10 (3.4%)	34 (5.0%)
Best response			
Complete response (CR)	215 (22%)	77 (26%)	138 (20%)
Partial response (PR)	50 (5.2%)	16 (5.5%)	34 (5.0%)
Stable disease (SD)	145 (15%)	50 (17%)	95 (14%)
Progressive disease (PD)	441 (46%)	120 (41%)	321 (48%)
Deceased at assessment	118 (12%)	30 (10%)	88 (13%)
Disease control rate (CR + PR+SD)	**410 (42%)**	**143 (49%)**	**267 (40%)**

Bold indicates the primary study outcome (disease control rate)

Continuous variables are presented as median (interquartile range); categorical variables as number (percentage). Stage reflects stage at initial diagnosis. Line of therapy reflects the treatment regimen number in which ICI was administered

*IQR* interquartile range, *ICI* immune checkpoint inhibitor, *CR* complete response, *PR* partial response, *SD* stable disease, *PD* progressive disease, *HEENT* head, ears, eyes, nose, throat

The primary outcome was DCR compared between time-of-day (TOD) infusion groups. DCR was defined as CR, PR, or SD per RECIST criteria as best response (Fig. 1A). In the overall cohort, early ICI administration was associated with a higher DCR compared with late ICI administration (49% vs. 40%; χ² *p* = 0.007). To confirm this finding was independent of potential confounders, we performed multivariable logistic regression adjusting for disease stage, age, and ethnicity. Early ICI administration remained independently associated with higher DCR (adjusted OR 1.18, 95% CI 1.02–1.38, *p* = 0.024). In propensity score-matched analyses (*n* = 472; 236 per group, well balanced on stage, age, and ethnicity), DCR was 49.2% in the early group versus 42.8% in the late group (OR 1.29, 95% CI 0.88–1.85, *p* = 0.17). The directional consistency between the primary and matched analyses is noteworthy; loss of statistical significance in the matched cohort reflects the substantial reduction in effective sample size rather than reversal of effect. Sensitivity analyses using three alternative exposure definitions (majority of infusions before noon, first infusion only, and median infusion time) yielded identical results (DCR 48.8% vs. 39.5%, *p* = 0.007 for all definitions), confirming that the exposure classification is robust in this dataset where each patient contributes one infusion time record. 

**Fig. 2:**
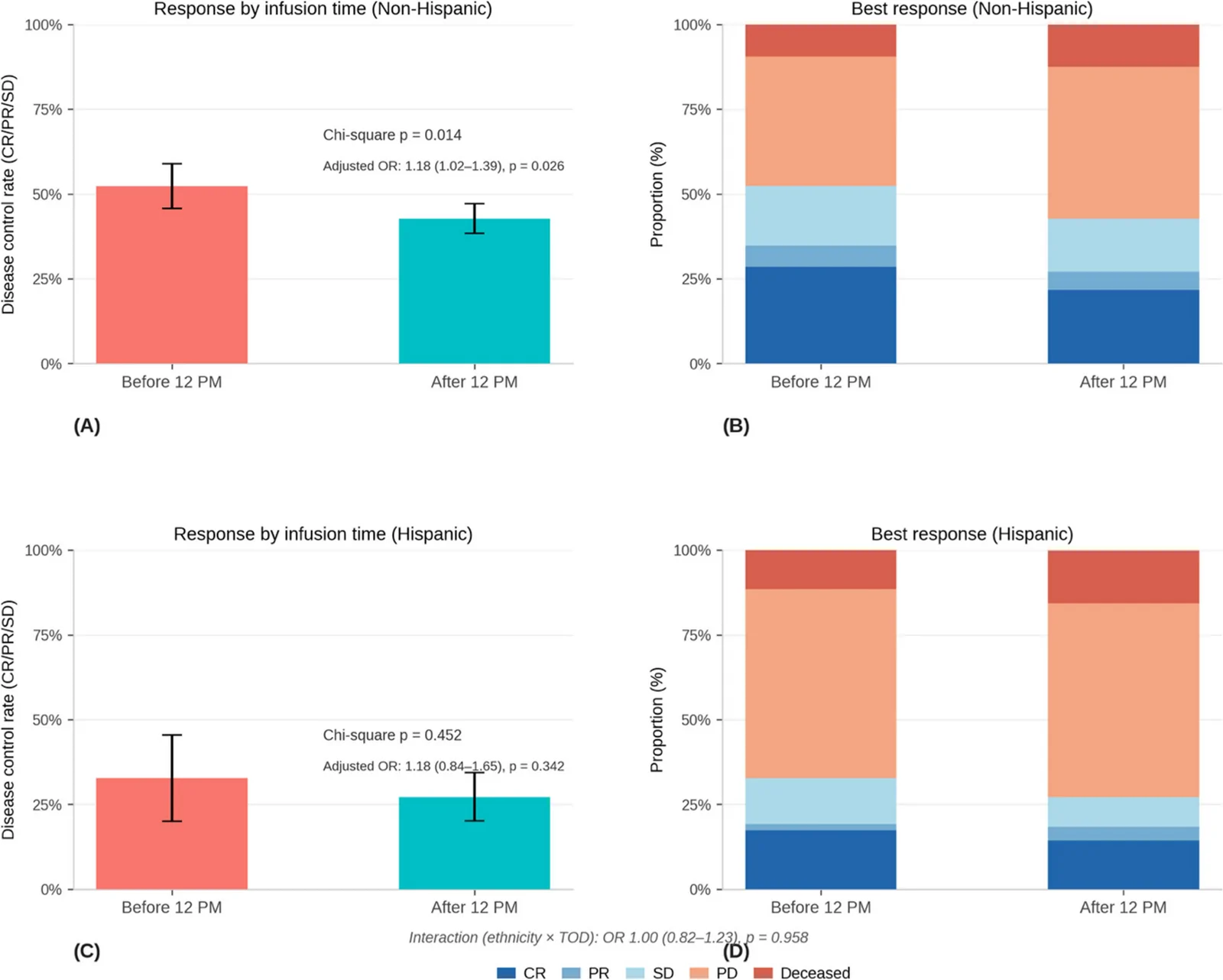
Association between time of immunotherapy administration and disease control rate by ethnicity. **A **DCR among non-Hispanic patients. Error bars indicate 95% CIs. P-value by chi-square test. Adjusted OR from logistic regression adjusted for stage and age. **B **DCR among Hispanic patients. Error bars indicate 95% CIs. Adjusted OR from logistic regression adjusted for stage and age. Interaction (ethnicity × TOD): OR 1.00 (0.82–1.23), *p* = 0.958; identical point estimates reflect a power difference rather than qualitative effect modification. CR = complete response; PR = partial response; SD = stable disease; OR = odds ratio; CI = confidence interval; TOD = time-of-day (**A**) Disease control rate (DCR = CR/PR/SD) according to infusion time among non-Hispanic patients. Error bars indicate 95% confidence intervals. P-value calculated using the chi-square test. Adjusted OR from multivariable logistic regression adjusted for stage and age. (**B**) Best response breakdown (CR/PR/SD/PD/Deceased) by infusion time among non-Hispanic patients shown as stacked proportions. (**C**) Disease control rate according to infusion time among Hispanic patients. Error bars indicate 95% confidence intervals. P-value calculated using the chi-square test. Adjusted OR from multivariable logistic regression adjusted for stage and age. (**D**) Best response breakdown by infusion time among Hispanic patients shown as stacked proportions. Interaction (ethnicity × TOD): OR 1.00 (0.82–1.23), p = 0.958; identical point estimates with divergent statistical significance reflect a power difference rather than qualitative effect modification. CR = complete response; PR = partial response; SD = stable disease; PD = progressive disease; OR = odds ratio; CI = confidence interval; TOD = time-of-day

Subgroup analyses demonstrated consistent directionality across subgroups (Supplementary Fig. S2). The TOD-DCR association was significant in males (early 50.9% vs. late 41.5%, *p* = 0.043) and directionally consistent in females (46.3% vs. 36.5%, *p* = 0.058). The effect was significant in patients aged 65 years or younger (47.7% vs. 35.6%, *p* = 0.010) and directionally consistent but attenuated in those older than 65 (50.0% vs. 44.4%, *p* = 0.275). By stage, the association was strongest in stage I–II (67.6% vs. 48.1%, *p* = 0.067) and consistent in direction across stage III and IV. By ICI agent, the association was significant for pembrolizumab (51.7% vs. 40.4%, *p* = 0.010) and directionally consistent for nivolumab (46.6% vs. 37.8%, *p* = 0.169); atezolizumab and durvalumab subgroups were underpowered for subgroup analysis. While we did not observe a significant association between the site of the primary tumor and TOD benefit, there was a notable trend toward increased relative benefit in the colorectal cohort, consistent with prior preclinical data [4,5] (Fig. 2).

**Fig. 3:**
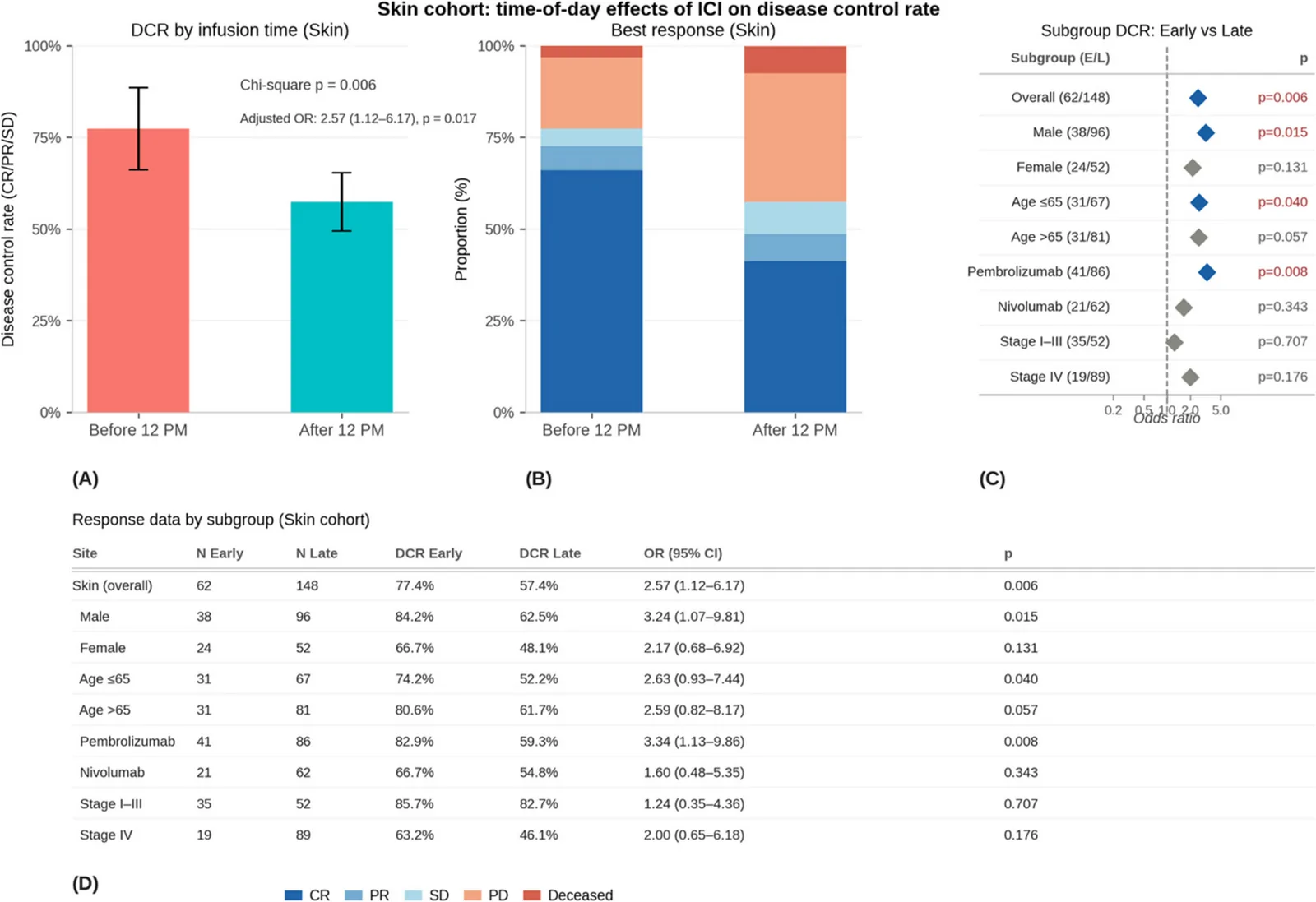
Skin cohort: time-of-day effects of immune checkpoint inhibitor administration on disease control rate. (**A**) Disease control rate (DCR = CR/PR/SD) according to infusion time (before 12 PM vs after 12 PM) in the skin cohort (n=210). Error bars indicate 95% confidence intervals. P-value calculated using the chi-square test. (**B**) Best response breakdown (CR/PR/SD/PD/Deceased) by infusion time in the skin cohort shown as stacked proportions. (**C**) Subgroup forest plot showing unadjusted odds ratios for DCR (early vs late ICI) within pre-specified demographic and treatment subgroups of the skin cohort. Points represent effect estimates; horizontal lines indicate 95% confidence intervals. The dashed vertical line represents no effect (OR = 1). (**D**) Data table summarizing response outcomes and odds ratios by subgroup within the skin cohort. CR = complete response; PR = partial response; SD = stable disease; PD = progressive disease; OR = odds ratio; CI = confidence interval; ICI = immune checkpoint inhibitor

Given that our catchment area includes a substantial Hispanic population, we next examined whether ethnicity modifies the association between ICI TOD and DCR (Fig. 3). This analysis was motivated by emerging evidence that ICI responsiveness may differ by ethnicity, potentially driven by population-level variation in HLA diversity and immunogenetic profiles [19–22], as well as a disproportionate lymphoid disease burden observed in this group [23,24]. In ethnicity-stratified analyses, earlier infusion time was associated with a higher DCR among non-Hispanic patients (52% vs. 43%, *p* = 0.014, adjusted OR 1.18, 95% CI 1.02–1.39) (Fig. 2A) but did not reach significance among Hispanic patients (32% vs. 27%, *p* = 0.452, adjusted OR 1.18, 95% CI 0.84–1.65) (Fig. 2B). Formal testing of the ethnicity-by-TOD interaction was non-significant (OR 1.00, 95% CI 0.82–1.23, *p* = 0.958), indicating that identical point estimates in both subgroups with divergent statistical significance reflects a power difference rather than a qualitative effect modification.

## Discussion

To our knowledge, this is the largest real-world study to date supporting the importance of ICI timing on tumor control. While prospective clinical data have demonstrated a beneficial role of early morning ICI administration [14], our study validates these findings in a large, heterogeneous real-world cohort spanning multiple primary tumor sites, stages, and demographics. Notably, no significant difference in the timing effect was observed based on tumor histology, and the benefit was consistent across both anti-PD-1 and anti-PD-L1 therapies.

These findings should be interpreted in the context of recent conflicting data. A patient-level meta-analysis of eight randomized trials in advanced NSCLC (ETOP-Roche i-TIMES) found no difference in OS by ICI administration time, whereas a randomized phase 3 trial conducted in an Asian NSCLC population reported significantly improved outcomes with morning administration [14]. One important consideration in reconciling these results is whether OS is the most appropriate endpoint for detecting a chronotherapeutic effect. Overall survival integrates a wide range of factors beyond direct tumor response, including subsequent lines of therapy, comorbidities, and competing causes of death, any of which may dilute a biologically real time-of-day effect on tumor-immune interactions. Radiographic disease control assessed per RECIST criteria more directly captures the immediate immunological consequence of drug administration timing and is not confounded by post-progression events, making DCR a more sensitive and interpretable endpoint for detecting the direct chronotherapeutic effect of ICI administration.

The modest adjusted OR of 1.18 warrants contextual discussion. An OR of 1.18 corresponds to an approximately 9-percentage-point difference in DCR (49% vs. 40%), which, from a zero-cost scheduling intervention, carries meaningful population-level implications. Sensitivity analyses using alternative exposure definitions yielded identical results, confirming robustness of the primary finding. However, when the model was extended to include ICI agent and line of therapy as additional covariates, the adjusted OR shifted to 1.31 (95% CI 0.96–1.77), with the confidence interval crossing 1. We attribute this attenuation partly to the methodological issue that line of therapy is a potential mediator of the TOD-outcome relationship rather than a pure confounder: sicker or more heavily pretreated patients may be scheduled later in the day, and adjusting for a mediator introduces collider bias that can attenuate the estimated effect. Similarly, in propensity score-matched analyses (*n* = 472), the OR was 1.29 (95% CI 0.88–1.85), directionally consistent with the primary analysis but non-significant, reflecting the substantial reduction in sample size. Taken together, these results support a real but modest chronotherapeutic effect that remains detectable in the primary adjusted analysis and directionally consistent across all sensitivity analyses, though its magnitude should be interpreted cautiously pending prospective validation.

Our ethnicity-stratified analysis revealed that the TOD benefit was confined to non-Hispanic patients, with no significant effect observed among Hispanic patients. One possible explanation is that Hispanic patients have been shown to harbor increased genomic instability in lymphoid lineage genes, conferring heightened susceptibility to certain lymphoid malignancies [23, 24]. Such differences in the lymphoid compartment may influence the circadian immune dynamics that underlie the chronotherapeutic benefit of morning ICI administration, though this hypothesis requires further investigation.

We did not formally report OS as a primary outcome, as there were imbalances between TOD groups in stage at treatment initiation, line of therapy, and tumor type. Because stage at diagnosis remains the most important prognostic factor for survival across nearly all solid tumors, these imbalances would significantly confound any survival analysis. In contrast, radiographic assessment of change in target lesion size per RECIST criteria is independent of tumor stage, making DCR a more reliable primary endpoint for this study. For completeness, a descriptive Kaplan–Meier OS analysis is presented as Supplementary Fig. S1; median OS was 27.4 months in the early group versus 21.0 months in the late group (log-rank *p* = 0.15). This non-significant OS result is consistent with the stage imbalance described above and does not contradict the DCR findings.

Limitations of this study include its retrospective single-center design, potential residual confounding despite multivariable adjustment, limited statistical power in the Hispanic and small-agent subgroups, absence of ECOG performance status and PD-L1 expression data in the pharmacy database, and a 2.3:1 imbalance between late and early patients reflecting real-world scheduling constraints. Further prospective studies are needed to validate these findings and to interrogate the underlying biologic mechanisms driving the observed chronotherapeutic effect.

## Conclusions

In a large, heterogeneous real-world cohort of patients with solid tumors, early ICI administration was independently associated with higher DCR after adjustment for disease stage, age, and ethnicity. The effect was directionally consistent across all sensitivity analyses, subgroup analyses, and the propensity score-matched cohort. These findings provide real-world support for a chronotherapeutic effect of ICI timing and identify DCR as a sensitive endpoint for its detection. Prospective validation across diverse tumor types and patient populations is warranted.

## Supplementary Information


Supplementary Material 1.


## Data Availability

Availability of data and materials: The datasets used and analyzed during the current study are available from the corresponding author on reasonable request.
