# Supplementary material for: Real-world evidence for the impact of circadian clock on the benefit from immune checkpoint inhibitors in solid tumors
Source: BMC Cancer. 2026 Jun 19;26:1030. doi: 10.1186/s12885-026-16364-w (PMC13523368; doi:10.1186/s12885-026-16364-w)
Supplement: Supplementary file 1 [file 12885_2026_16364_MOESM1_ESM.docx]

**Supplementary Fig. S1.** Kaplan–Meier overall survival estimates by infusion time. Median OS: 27.4 months (before 12 PM) vs 21.0 months (after 12 PM); log-rank p = 0.15. Presented descriptively; stage imbalance between TOD groups substantially confounds this analysis (see Methods). Number at risk shown below x-axis.

**Supplementary Fig. S2.** Subgroup analyses of early versus late ICI infusion on disease control rate. Unadjusted ORs with 95% bootstrap CIs shown for pre-specified subgroups. Atezolizumab and durvalumab subgroups (n = 39 and n = 27, respectively) were underpowered and are not shown. OR = odds ratio; CI = confidence interval.
